# Medical Opinion and Movement

**Published:** 1911-02-04

**Authors:** 


### Medical Opinion and Movement.

### Surgery of the Obese.

In a contribution to the *Monthly Cyclopedia and Medical Bulletin* Dr. Robert T. Morris, of New York, gives some useful suggestions and hints in regard to special precautions that should be taken by the surgeon when performing an operation upon patients possessed of an excess of adipose tissue. Very clean incisions should be made through the fatty tissues so as to cause as few fatty tabs and irregularities as possible. He advises avoiding the use of artery forceps, because they crush a certain amount of tissue which must be disposed of by the lymphatics, and the lymphatics of adipose tissue are neither abundant nor active. The arteries should be secured by slipping a ligature under each vessel by means of a needle, and as little fat as possible must be included in the ligature, as the fat is liable to slip out afterwards, leaving the ligature loose in the course of an hour or so. On this account also the ligatures must be secured firmly, or, as the American expresses it, "snugly." The free oil that collects in the wound should not be wiped away with the blood, as any such procedure injures the adipose tissue and liberates more oil. The author recommends, therefore, that the wound be irrigated continually with normal salt solution, so as to float away the excess of oil in the solution. As there is frequently fatty degeneration of the muscular tissues, suturing of the muscles should be avoided as far as possible. This can be done by avoiding incisions through the muscle belly, if possible, and by suturing the muscle sheaths, without passing the sutures into the muscle itself. Similarly, in the closure of the wound no sutures should pass through the subcutaneous fatty tissues.

### Flat Foot.

In 1902 Nicoladoni first described his operation for flat foot, which consists in division of the tendo Achillis, the upper end being then tucked in upwards under the fascia and sutured in this position. Nicoladoni argued that since the calf-muscles are opponents to the short plantar flexors, division of the tendo Achillis would cause the plantar muscles to functionate more actively, resulting in the return of the foot towards the normal. He reported on five cases of severe flat foot, in which the operation has been successfully performed. Dr. J. Hertle, of Graz, now reports further on the operation in the *Archiv für Klinische Chirurgie*. He has also obtained very satisfactory results by this method of treatment. From three to six weeks after operation the patients are able to return to their occupation. Of all the operative procedures for this condition Nicoladoni's is, in his opinion, the simplest and relieves pain in the shortest time. It tends to induce a return of the foot to its normal anatomical structure. Severe disturbances in gait as a result of the operation are only temporary. Despite the method of treating the Achillis tendon a new formation of tendon tissue takes place after a time, and the calf-muscles again functionate normally.

### Carcinoma and Radium.

In the *Munchener Medizinische Wochenschrift* Dr. A. Exner, of Vienna, reports four cases of carcinoma which have been apparently cured permanently by treatment with radium. The first patient was a woman, aged forty-two, with a small carcinoma of the cheek. A single exposure of one half-hour caused the disappearance of the growth in fourteen days. This was seven years ago, and it has not returned. A second patient was a woman, aged seventy-three, with a tumour of the cheek involving the mouth and slightly adherent to the upper and lower jaws. She was treated for four months and has also remained well for seven years. The third case was that of a man aged seventy-seven, who was operated upon for tumour of the upper jaw involving the antrum. Parts of the neoplasm could not be removed and were therefore exposed to the radium emanations for fourteen days. There was no recurrence of the growth up to the patient's death, two years later, from pneumonia. The last patient was a man, aged fifty-eight years, with a carcinoma involving the whole of the upper lip and infiltrating the neighbouring tissues. For four months interrupted exposures were given, the severe reactions necessitating intermissions. The patient then refused further treatment, but at this time a small piece of the neoplasm was excised, and examination showed it to be carcinoma, the vitality of which was said to be much impaired by the treatment. The patient is still well four and a half years later. Thus all the growths were epithelial in origin and quite superficial, a form which is especially amenable to the treatment with radium.

### Post-operative Vesical Trouble.

Temporary paralysis of the bladder, resulting in retention of urine, is a frequent occurrence after abdominal operations. Instead of resorting to the usual method of treatment by catheterism, which is more or less painful to the patient, and carries with it a certain risk of infection, Dr. O. Franck, of Frankfort, recommends the injection of a small amount of glycerine and boric acid. He finds that such an injection excites peristalsis of the bladder and produces a spontaneous evacuation in a short time. Moreover, in the majority of cases, the effect is definite and lasting, so that only exceptionally the injection must be repeated. Dr. Franck has employed this method of treatment in a large number of cases and has always found it successful. At first he injected into the bladder about 30 c.c. of 2-per-cent. boracic glycerine through a catheter, but more recently he has found it sufficient simply to inject a smaller amount, usually 15 to 20 grammes, into the meatus. A large amount of this runs back, so that only 5 to 10 grammes of the glycerine enter the bladder, but this is quite sufficient to effect the desired result, and spontaneous micturition takes place in the course of about twenty minutes. The author has also applied the treatment to other conditions of retention of urine, and has obtained successful results in bladder paralysis of nervous or mechanical origin, the glycerine acting as a sort of fluid catheter. Even in cases of retention due to stricture or prostatic hypertrophy the method gives relief, although of course only temporary, by inducing an evacuation. The treatment appears to be especially indicated, however, in cases of retention of a temporary and more or less obscure nature, and also for patients who do not lend themselves easily to catheterism, but it is contra-indicated in conditions of acute inflammation of the anterior part of the urethra.

### Professor Galle's Discovery.

An editorial in the *Journal of Tropical Medicine and Hygiene* announces that Professor Gallé, of Rome, has communicated to Professor Ronald Ross his discovery of the Leishman-Donovan parasite in a case of "ponos." The latter is a curious disease which occurs sporadically in Cyprus and certain islands off the coast of Greece. From one of the latter, Spezzia, comes the patient in whom the parasite has been discovered. This discovery, if confirmed, will show that kala-azar actually exists in the Mediterranean; or at any rate some disease very closely akin to kala-azar. It is interesting to recall that the possibility of "ponos" and kala-azar being one and the same occurred to Sir Patrick Manson four years ago, and has received emphasis from several recent researches and discoveries. The term Leishmaniasis now includes kala-azar, ponos, and Oriental sore; and it is suggested that perhaps cancrum oris is due to the same infective agent. In temperate latitudes cancrum oris is a disease of young children only, but in the tropics it is much commoner, is found at all ages, and is frequently associated with the presence of Leishman-Donovan bodies. It is time, says the writer in the *Journal*, that the pathology and ætiology of cancrum oris in northern Europe were tested from these points of view. The speculation thus advanced is ingenious and well worthy of investigation.

### The Bacteriology of Scarlet Fever.

That the infection of scarlet fever is harboured in the throat and nasal passages, and not in the scales during the period of desquamation, has long been the belief of Dr. Schultze, who reports in the *Medical Record* his researches into the bacteriology of the fauces and post-nasal fossæ of patients suffering from this disease. In 555 cultures taken from the latter region in undoubted cases of scarlet fever he has found a coccus in 459 which has certain constant characters. Care must be taken to swab the post-pharyngeal wall, not the tonsils, when examining the case. The same organism has been recovered from the serum of a papule in a fatal case of scarlet fever, from the blood, and from the pus in five cases complicated by otitis media. On the other hand, the desquamating scales do not contain it. In many cases streptococci of various kinds are also found, and have been cultured from the heart, etc., in fatal cases; but no disease resembling scarlet fever has ever been reproduced from them in animals or human beings. Five pigs have been inoculated with this coccus of Schultze, and all developed rash, fever, and malaise. Further investigations along these lines are being carried out. The author further notes that 10 per cent. of clinically undoubted scarlet-fever patients harbour the Klebs-Löffler bacillus; and a fine short streptococcus is also quite common, as well as the micrococcus which he suggests as the specific organism of scarlet fever. One hundred consecutive examinations of the pharynx of healthy children who had never had scarlet fever failed to produce a single coccus with the characteristics of this micrococcus; and the same result followed when a hundred examinations were made of children suffering from measles, erysipelas, diphtheria, chicken-pox, influenza, and similar diseases. In a subsequent series the coccus was found in fifty out of fifty-five scarlatina and in three out of fifty non-scarlatina cases.

### The Treatment of Eclampsia.

The peculiar prevalence of eclampsia in Scotland has been emphasised recently by Dr. Haultain (see The Hospital, January 21, 1911, p. 491). It is worth while, therefore, to note the trend of Scottish obstetrical practice in the treatment of this troublesome condition. In the *Journal of Obstetrics and Gynæcology of the British Empire* Dr. Ballantyne describes his experiences in the autumn quarters of the past six years, during each of which he has had charge of the Edinburgh Royal Maternity Hospital. In the first three years of this period (1905-6-7) he had twelve cases of eclampsia with three maternal and nine fœtal deaths. In the later three (1908-9-10) he had twenty-six cases, with but two maternal and eleven fœtal deaths. This vast improvement has coincided with the abandonment of *accouchement forcé*. In 1908-10 labour was hardly ever expedited, whereas in the earlier years it was so very frequently. Morphia and thyroid extract also have been less used of late; venesection and transfusion, however, have been more extensively employed. Whether eclampsia develops during pregnancy, in labour, or in the puerperium, the author's routine is now venesection with subsequent saline transfusion; washing out the stomach with bicarbonate of soda solution and leaving in it a large dose of magnesium sulphate, a copious enema of soap and water and castor oil, and a hot pack. Chloroform is used to control convulsions. Obstetric interference may be, he thinks, still sometimes justifiable, but would be contingent upon circumstances which of late he has not often met with.

### Sanitary Arrangements on Railway Trains.

Dr. Rapin, of Nantes, in *L'Hygiène Générale et Appliquée* draws attention to a danger to which the present type of water-closets provided in railway carriages may give rise in the presence of a cholera epidemic. The ordinary system in vogue on most railways is a pan with a hole in the bottom communicating directly with the ground. This he considers not only primitive, but positively dangerous. Fæcal material is allowed to be washed down this hole when the train is travelling at a high speed, with the result that it is broken up and blown about by the wind caused by the train and scattered far and wide on to the wheels of the train and into the hedges and ditches, whence it is bound to find its way eventually to ponds and other collections of water. The effect of such a thing happening with material infected by the bacillus of cholera can be easily imagined, for the organisms can live a long time in water. The author would, therefore, have it made compulsory for railway closets to be connected with a metal receptacle, in which some antiseptic fluid has already been placed. This receptacle should be cleaned out daily, and the chance of infection in this way eliminated.

### Tetany in the Infant.

In a recent *Thèse de Paris* Jardel attempts to classify the signs and symptoms peculiar to tetany in the infant, and to review the principal affections with which it is likely to be confounded. The disease may take on any of three principal varieties, the atypical (or tetanoid state), the idiopathic persistent, and the pseudo-tetanic. Whatever be its form, tetany has for its cardinal symptoms (1) contractures or tonic convulsions characterised by Trousseau's sign; (2) convulsions (of clonic type and eclampsia); (3) mechanical hyperexcitability of the nerves (especially the facial or Chvostek's sign); (4) electrical hyperexcitability of the nerves. Secondary signs also exist, such as vaso-motor and trophic troubles, pains, polyuria, etc. In every young child tetany may be atypical, latent, and at times febrile. In the new-born child it must be differentiated from tetanus. The latter usually results from injury, is accompanied by fever, trismus, and opisthotonos, and lacks Chvostek's sign. Electrical examination may furnish further evidence. In tetany galvanic excitability of nerves is increased, but not in tetanus. Tetany may also be confounded with meningeal affections, such as meningeal hæmorrhage, tuberculous cerebro-spinal and chronic meningitis. Lumbar puncture will give useful information when diagnosis is in doubt. Tetany in young children is more easily distinguished from sclerema neonatorum and uræmic encephalopathy, but it is, according to the author, still doubtful whether it is to be distinguished from that half-physiological, half-pathological condition called by Hochsinger myotonia neonatorum.

### Exostoses of the Scapula.

As the result of a case of exostosis of the shoulder-blade observed and operated on by Delépine, this author has published a study of this affection in the *Journal des Sciences Médicales de Lille*. He has been able to find twenty-seven other cases in which an operation was performed. These tumours are met with during the period of growth, and especially in males. Rickets and trauma are predisposing factors. They arise from the cartilage near the neck or in the region of the spinal border or from the inferior angle of the bone. In structure they are similar to bone, and their volume is generally in inverse proportion to their number. They give rise to symptoms which vary according to their locality. When on the anterior surface of the scapula they push the bone backwards, interfere with movements of the shoulder, and may produce intercostal neuralgia. When situated posteriorly they produce an elevation over which the skin moves freely; the tumour is fixed to the bone, but causes no pain. There is no effect on the general health. Development is progressive, prognosis good; degeneration may occur, however. Differential diagnosis between such a tumour and callus, tumours of the soft parts, purulent collections, malignant tumours, etc., is easy, but it is more difficult to differentiate the lump from a chondroma. In removing the exostosis a larger portion of the scapula should be cut away than the actual point of attachment of the tumour to the bone.

### Table Jellies.

In the Journal of the *Royal Army Medical Corps* Major Beveridge points out the great value of meat and fruit jellies as articles of invalid diet. Gelatine itself, he remarks, is a protein sparer to an even greater degree than are fats and carbohydrates. Besides these protein-sparing qualities, gelatine is especially useful to the organism when in a poor state of health, for persons in a condition of low nitrogenous equilibrium profit especially by taking gelatine. Moreover, the latter is easy of digestion and has the power of fixing any excess of acidity in the stomach. Again, jellies contain a large amount of sugar, which is a true food; and the whole combination gives rise to considerable heat and energy. It is, therefore, not at all necessary to wait for convalescence before ordering jellies in most cases. After considering in detail the different forms in which jellies are marketed—in bottles, in tablets, and as granulated powders—the author expresses his belief in the superiority of that made direct from calves' feet and eaten fresh. He defines the essentials of a good table jelly as six in number. It must set at ordinary temperatures (or in the tropics on ice); to secure this there must be at least 1 per cent. of gelatine, and there must be only very slight acidity. It must be as clear and transparent as possible to tempt the appetite best. It must be palatable and sharp to the taste; flavouring agents are admissible for this purpose. It must contain about 80 per cent. of sugar. It must not contain any preservative, nor any albumin. Any colouring matter must be of a harmless kind, such as burnt sugar, saffron, cochineal. To these essentials may be added a seventh. The jelly must be supplied in an easily portable form in a casing which resists atmospheric changes. Ordinarily the paper packets in which calves' feet jellies are supplied are useless for the purpose of keeping the contents dry or preventing "shaling" in the jelly square.

